# Supplementary material for: Global increase in tropical cyclone rain rate
Source: Nat Commun. 2021 Sep 9;12:5344. doi: 10.1038/s41467-021-25685-2 (PMC8429722; doi:10.1038/s41467-021-25685-2)
Supplement: Supplementary file 1 — Supplementary Information [file 41467_2021_25685_MOESM1_ESM.pdf]

Supplementary Materials

Global Increase in Tropical Cyclone Rain Rate

Oscar Guzman and Haiyan Jiang*

Department of Earth & Environment, Florida International University, Miami, Florida

Submitted to Nature: July 28, 2020

Revised on April 5, 2021
Final revision on May 12, 2021

* Corresponding author address: Dr. Haiyan Jiang, Department of Earth and Environment, Florida International University, AHC5 room 371, 11200 SW 8th Street, Miami, FL 33199. Phone: 305-348-2984. E-mail: haiyan.jiang@fiu.edu

Supplementary materials:

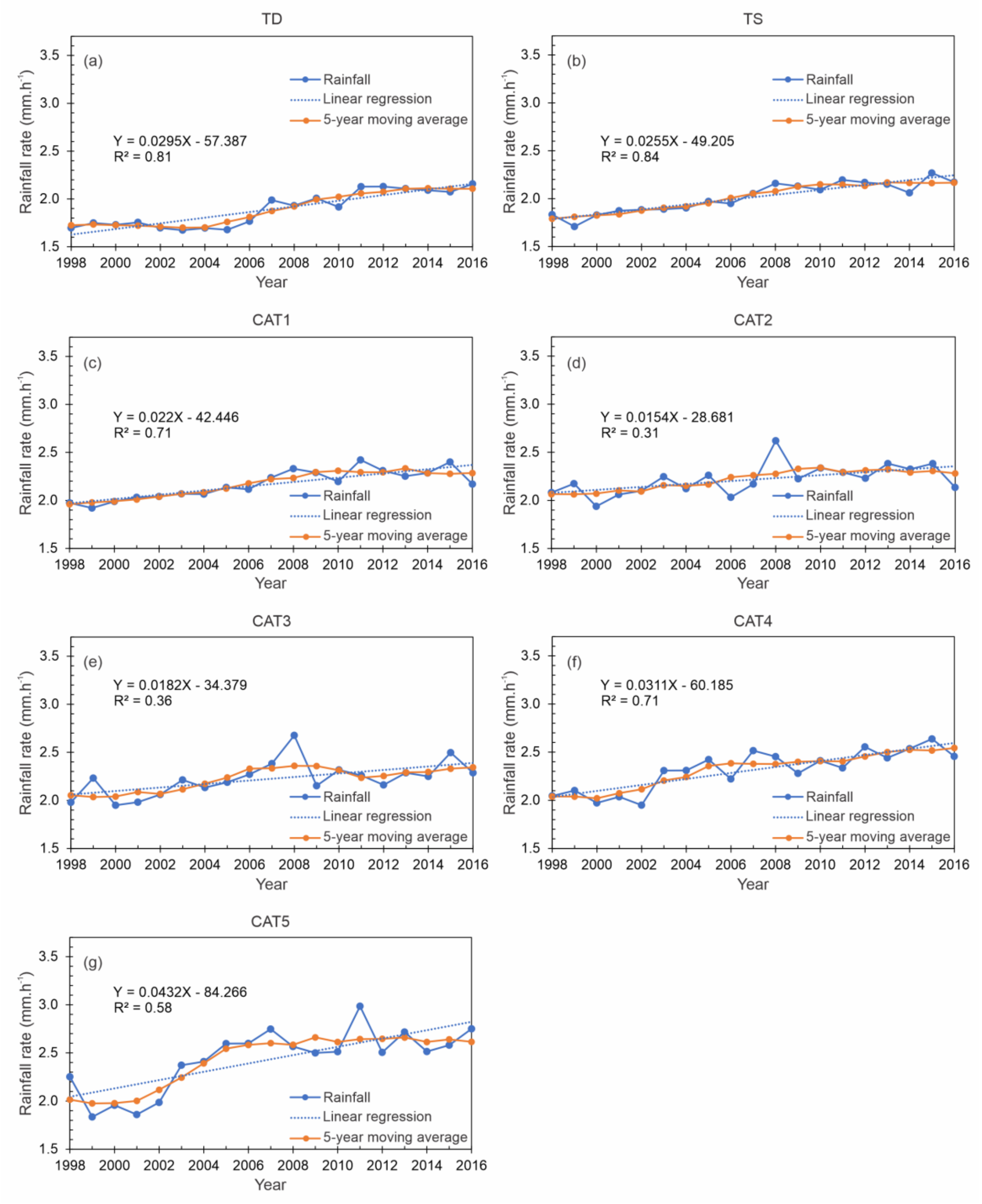

Supplementary figure 1. Time series and the linear regression fit of TC rain averaged within 0-500km around the storm center for different TC intensity categories: (a)TD, (b)TS, (c) CAT1, (d) CAT2, (e) CAT3, (f)CAT4, and (g) CAT5.

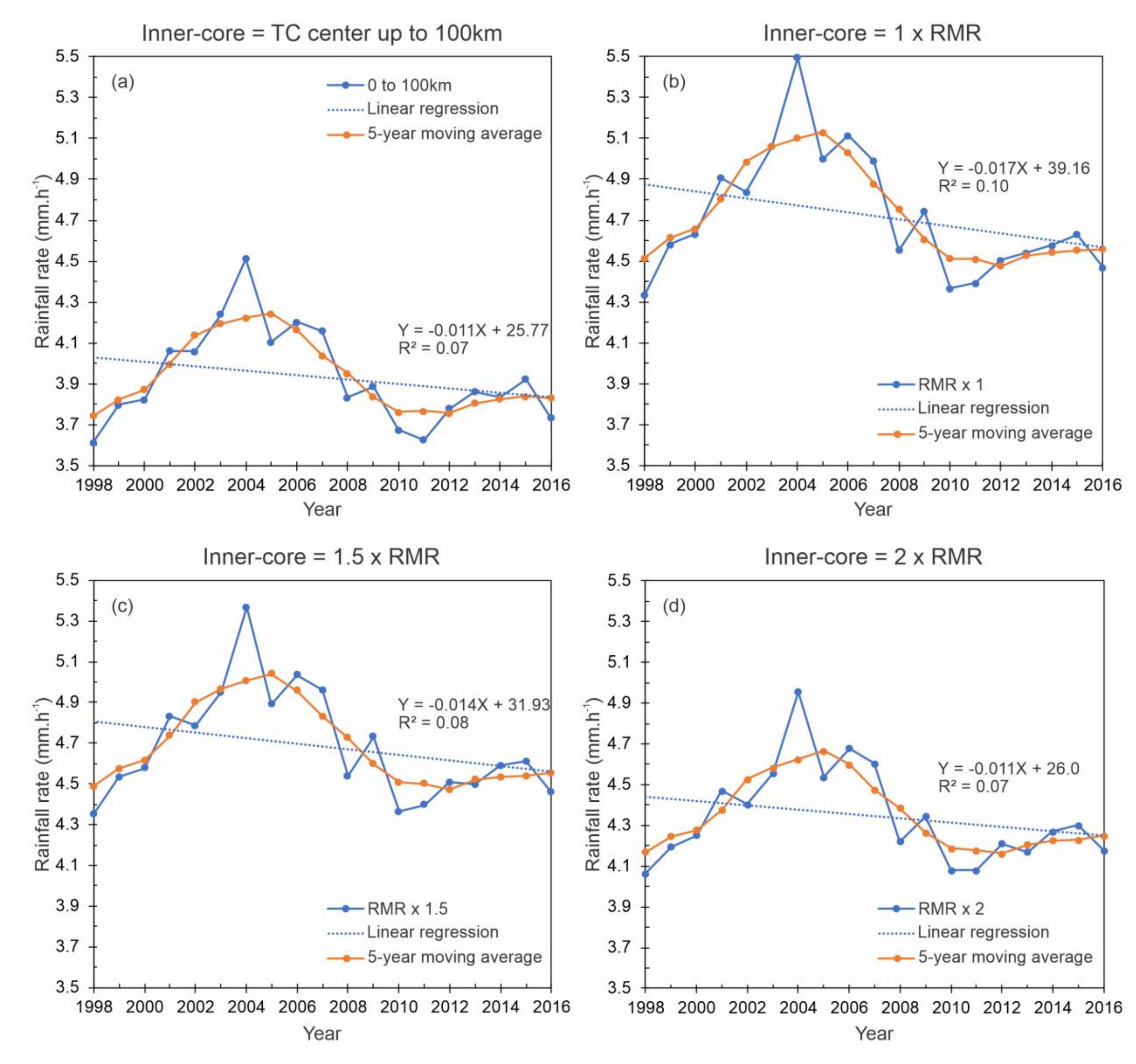

Supplementary figure 2. Time series and the linear regression fit of TC rain averaged within the inner-core for TCs in all global TC-prone basins. Figure shows a sensitivity test between different methods to define the inner-core extent from the TC center as a function of the radius of maximum rainfall. (a) From 0 to 100 km, (b) within 1x RMR, (c) within 1.5 x RMR, and (d) within 2 x RMR. The linear fitting function and square of the correlation R are indicated. The 5-year moving average is also shown.

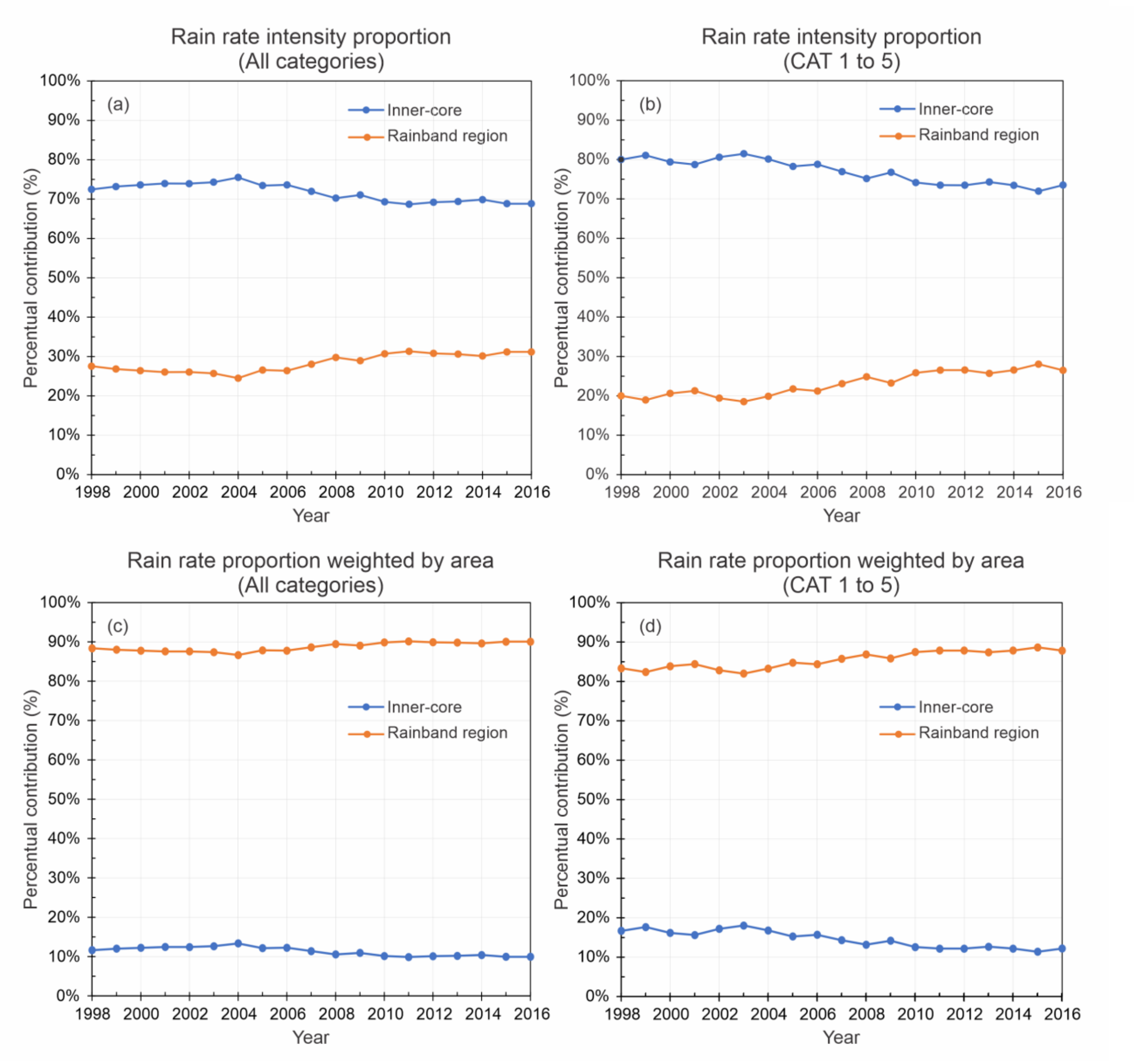

Supplementary figure 3. Time series showing the approximated global rainfall contributions of the inner-core and the rainband region. (a) rain rate intensity proportion for all storm categories, (b) rain rate intensity proportion for only storms from category 1 to 5, c) rain rate proportion weighted by area for storm categories, and d) rain rate proportion weighted by area for storm from category 1 to 5. Values presented as percentage.

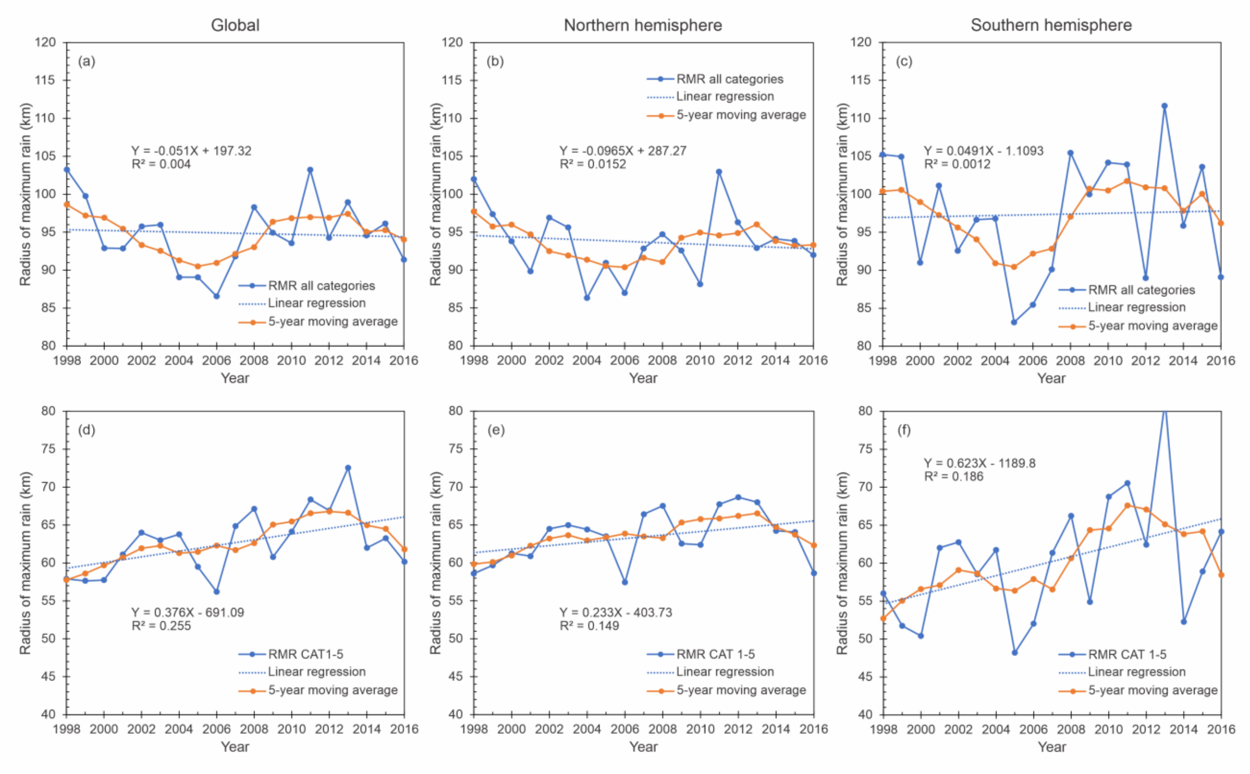

Supplementary figure 4. Time series and the linear regression fit of TC rain averaged within 0-500km around the storm center for (a) radius of maximum rain globally, (b) radius of maximum rain in the northern hemisphere, and (c) radius of maximum rain in the southern hemisphere, (d) radius of maximum rain globally for categories 1-5, (e) radius of maximum rain in the northern hemisphere for categories 1-5, and (f) radius of maximum rain in the southern hemisphere for categories 1-5.

Supplementary table 1: Percentage of change in yearly trends for mean TC rainfall (0-500km TCPF method), TC inner-core rain, and rainband rain in all TC basins. Values in mm.h^{-1} and percentage.

	Total				Inner-core (All categories)				Inner-core (CAT1 to CAT5)				Outer rainband			
Year	Raw	% of change	Regression	% of change	Raw	% of change	Regression	% of change	Raw	% of change	Regression	% of change	Raw	% of change	Regression	% of change
1998	1.82		1.876		4.06		4.426		6.87		7.242		1.55		1.491	
1999	1.81	-0.8%	1.903	1.4%	4.20	3.1%	4.415	-0.2%	7.26	5.4%	7.146	-1.3%	1.54	-0.4%	1.516	1.6%
2000	1.82	0.9%	1.930	1.4%	4.25	1.3%	4.404	-0.2%	6.19	-17.3%	7.050	-1.4%	1.53	-0.8%	1.540	1.6%
2001	1.87	2.5%	1.957	1.4%	4.47	4.9%	4.393	-0.2%	6.27	1.2%	6.954	-1.4%	1.57	3.1%	1.564	1.6%
2002	1.86	-0.6%	1.984	1.4%	4.40	-1.5%	4.382	-0.2%	6.88	9.0%	6.858	-1.4%	1.55	-1.4%	1.589	1.5%
2003	1.88	1.1%	2.012	1.4%	4.56	3.4%	4.372	-0.2%	7.65	10.0%	6.761	-1.4%	1.58	1.5%	1.613	1.5%
2004	1.93	2.8%	2.039	1.3%	4.95	8.1%	4.361	-0.2%	7.25	-5.5%	6.665	-1.4%	1.61	2.0%	1.638	1.5%
2005	1.98	2.3%	2.066	1.3%	4.54	-9.2%	4.350	-0.2%	6.68	-8.5%	6.569	-1.5%	1.64	2.0%	1.662	1.5%
2006	1.97	-0.5%	2.093	1.3%	4.68	3.0%	4.339	-0.2%	6.77	1.2%	6.473	-1.5%	1.68	2.1%	1.686	1.4%
2007	2.11	6.5%	2.120	1.3%	4.60	-1.6%	4.328	-0.2%	6.62	-2.2%	6.377	-1.5%	1.79	6.5%	1.711	1.4%
2008	2.17	2.8%	2.148	1.3%	4.22	-9.1%	4.318	-0.3%	6.12	-8.2%	6.280	-1.5%	1.79	-0.3%	1.735	1.4%
2009	2.12	-2.1%	2.175	1.3%	4.34	2.9%	4.307	-0.3%	6.31	3.0%	6.184	-1.6%	1.77	-1.1%	1.760	1.4%
2010	2.09	-1.5%	2.202	1.2%	4.08	-6.5%	4.296	-0.3%	5.71	-10.5%	6.088	-1.6%	1.81	2.2%	1.784	1.4%
2011	2.22	5.6%	2.229	1.2%	4.08	0.0%	4.285	-0.3%	5.66	-0.9%	5.992	-1.6%	1.86	2.9%	1.808	1.3%
2012	2.20	-0.9%	2.256	1.2%	4.21	3.1%	4.274	-0.3%	5.47	-3.5%	5.896	-1.6%	1.87	0.6%	1.833	1.3%
2013	2.18	-0.7%	2.284	1.2%	4.17	-0.9%	4.264	-0.3%	5.67	3.6%	5.799	-1.7%	1.84	-1.9%	1.857	1.3%
2014	2.15	-1.3%	2.311	1.2%	4.27	2.3%	4.253	-0.3%	5.74	1.2%	5.703	-1.7%	1.84	0.1%	1.882	1.3%
2015	2.29	6.1%	2.338	1.2%	4.30	0.7%	4.242	-0.3%	5.45	-5.4%	5.607	-1.7%	1.95	5.4%	1.906	1.3%
2016	2.20	-4.5%	2.365	1.2%	4.18	-2.9%	4.231	-0.3%	5.47	0.3%	5.511	-1.7%	1.89	-2.9%	1.930	1.3%
Yearly % of change (mean)		1.0%	-	1.3%	-	0.1%	-	-0.2%		-1.5%		-1.5%	-	1.1%	-	1.4%
Total % of change(mean)		20.6%	-	26.1%	-	2.8%	-	-4.4%		-20.4%		-23.9%	-	22.5%	-	29.5%

Supplementary table 2: Summary of statistics of trends for mean TC inner-core rain, rainband rain, and radius of maximum rainfall in all TC basins.

	Linear regression		Mann-Kendall trend test			Sen's slope		
	Equation	R-squared	Kendall's tau	p-value alpha =0.05	Test interpretation	Sen's slope	Lower bound for 95% confidence	Upper bound for 95% confidence
From 0 to 500 km truncation	y = 0.0386x - 75.459	R^2 = 0.925	0.81	< 0.0001	There is a trend in the series	0.037	0.031	0.043
From 0 to 500 km TCPF method	y = 0.0272x - 52.470	R^2 = 0.903	0.80	< 0.0001	There is a trend in the series	0.027	0.022	0.031
Inner-core global all categories	y = -0.0108x + 26.004	R^2 = 0.066	-0.111	0.534	There is no trend in the series	-0.008	-0.035	0.013
Inner-core global categories 1-5	y = -0.0962x + 199.45	R^2 = 0.629	-0.626	< 0.0001	There is a trend in the series	-0.101	-0.145	-0.058
Rainband region all categories	y = 0.0244x - 47.26	R^2 = 0.9272	0.86	< 0.0001	There is a trend in the series	0.024	0.021	0.029
Rainband region all categories 1-5	y = 0.0250x - 48.268	R^2 = 0.8098	0.673	< 0.0001	There is a trend in the series	0.026	0.018	0.033
RMR global all categories	y = -0.051x + 197.32	R^2 = 0.0041	-0.029	0.890	There is no trend in the series	-0.049	-0.496	0.394
RMR global categories 1-5	y = 0.3756x - 691.09	R^2 = 0.255	0.345	0.041	There is a trend in the series	0.368	0.022	0.805

Supplementary table 3: Summary of statistics of trends for mean TC rain (0-500 km using TCPF method) in different 6 different TC basins.

	Linear regression		Mann-Kendall trend test			Sen's slope		
	Equation	R-squared	Kendall's tau	p-value alpha =0.05	Test interpretation	Sen’s slope	Lower bound for 95% confidence	Upper bound for 95% confidence
North Atlantic	y = 0.0405x - 79.045	$R^2 = 0.748$	0.67	< 0.0001	There is a trend in the series	0.042	0.027	0.050
E&C Pacific	y = 0.0169x – 32.228	$R^2 = 0.454$	0.52	0.00011	There is a trend in the series	0.015	0.007	0.029
North West Pacific	y = 0.0370x - 72.072	$R^2 = 0.842$	0.79	< 0.0001	There is a trend in the series	0.036	0.030	0.042
North Indian Ocean	y = 0.0317x - 61.344	$R^2 = 0.513$	0.51	0.00186	There is a trend in the series	0.030	0.016	0.051
South Indian Ocean	y = 0.0190x - 36.177	$R^2 = 0.592$	0.61	0.00011	There is a trend in the series	0.021	0.014	0.027
South Pacific Ocean	y = 0.0177x - 33.615	$R^2 = 0.443$	0.47	0.00405	There is a trend in the series	0.017	0.006	0.026

Supplementary table 4: Summary of statistics of trends for mean TC rain (0-500 km using TCPF method) in different 7 different TC intensity categories.

	Linear regression		Mann-Kendall trend test			Sen's slope		
	Equation	R-squared	Kendall's tau	p-value alpha =0.05	Test interpretation	Sen's slope	Lower bound for 95% confidence	Upper bound for 95% confidence
Tropical depression	y = 0.0295x - 57.387	R^2 = 0.808	0.60	0.00016	There is a trend in the series	0.028	0.020	0.036
Tropical storm	y = 0.0255x - 49.205	R^2 = 0.845	0.79	< 0.0001	There is a trend in the series	0.026	0.019	0.032
Category 1	y = 0.0222x - 42.446	R^2 = 0.712	0.68	< 0.0001	There is a trend in the series	0.024	0.018	0.030
Category 2	y = 0.0154x - 28.681	R^2 = 0.307	0.47	0.00405	There is a trend in the series	0.015	0.007	0.026
Category 3	y = 0.0182x - 34.379	R^2 = 0.336	0.48	0.00315	There is a trend in the series	0.019	0.006	0.031
Category 4	y = 0.0311x - 60.185	R^2 = 0.705	0.63	< 0.0001	There is a trend in the series	0.031	0.020	0.043
Category 5	y = 0.0432x - 84.266	R^2 = 0.575	0.58	0.00031	There is a trend in the series	0.040	0.016	0.062

Supplementary table 5: Summary of trend analyses of seas surface temperature, total precipitable water, and radius of maximum wind speed. Values comparing northern and southern hemispheres.

	Linear regression		Mann-Kendall trend test			Sen's slope		
	Equation	R-squared	Kendall's tau	p-value alpha =0.05	Test interpretation	Sen's slope	Lower bound for 95% confidence	Upper bound for 95% confidence
SST (NH)	y = 0.0152x - 2.2692	$R^2 = 0.2487$	0.258	0.029	There is a trend in the series	0.016	0.002	0.032
SST (SH)	y = -0.0038x + 35.944	$R^2 = 0.0083$	-0.029	0.890	There is no trend in the series	-0.001	-0.027	0.019
TPW (NH)	y = 0.1056x - 153.77	$R^2 = 0.3129$	0.415	0.013	There is a trend in the series	0.124	0.036	0.193
TPW (SH)	y = -0.0126x + 82.537	$R^2 = 0.0053$	-0.076	0.679	There is no trend in the series	-0.014	-0.119	0.065
VMAX (NH)	y = -0.1202x + 290.04	$R^2 = 0.0736$	-0.111	0.534	There is no trend in the series	-0.077	-0.370	0.146
VMAX (SH)	y = 0.1755x - 304.70	$R^2 = 0.0448$	0.006	0.990	There is no trend in the series	0.006	-0.232	0.439

Supplementary table 6: Number of best track records (3B42 observations) for TCs during 1998-2016 in different TC intensity categories and different TC-prone basins.

Category/Basin	ATL	ECPA	NWP	NIO	SIO	SPA	Total
Tropical Depression	2,914	4,537	7,809	1,728	6,425	2,423	25,836
Tropical Storm	6,336	6,348	8,695	1,635	6,519	2,861	32,394
Category 1	2,073	1,732	3,438	264	1,746	767	10,020
Category 2	760	800	1,854	69	795	403	4,681
Category 3	589	642	1,506	101	800	344	3,982
Category 4	476	410	1,478	78	598	260	3,300
Category 5	125	64	595	33	97	115	1,029
Total	13,273	14,533	25,375	3,908	16,980	7,173	81,242

Supplementary table 7: Number of best track records (3B42 observations) for TCs during 1998-2016 in different years and different TC-prone basins.

Year/Basin	ATL	ECPA	NWP	NIO	SIO	SPA	Total
1998	850	956	1,050	310	1,271	745	5,182
1999	829	645	1,468	240	1,031	436	4,649
2000	709	922	1,623	175	1,285	340	5,054
2001	712	714	1,764	192	897	346	4,625
2002	615	869	1,803	153	989	249	4,678
2003	830	679	1,708	227	1,395	476	5,315
2004	908	539	1,820	247	1,059	192	4,765
2005	1,290	691	1,227	256	734	370	4,568
2006	537	880	1,274	129	729	375	3,924
2007	398	544	952	192	940	314	3,340
2008	812	678	992	229	1,154	201	4,066
2009	296	755	1,289	114	857	284	3,595
2010	842	352	701	202	558	516	3,171
2011	781	533	1,181	179	633	393	3,700
2012	916	642	1,354	112	899	268	4,191
2013	389	664	1,240	342	943	315	3,893
2014	316	1,161	1,080	209	617	384	3,767
2015	459	1,370	1,735	188	632	526	4,910
2016	784	939	1,114	212	357	443	3,849
Total	13,273	14,533	25,375	3,908	16,980	7,173	81,242
